# Improving a genetically encoded voltage indicator by modifying the cytoplasmic charge composition

**DOI:** 10.1038/s41598-017-08731-2

**Published:** 2017-08-15

**Authors:** Sungmoo Lee, Tristan Geiller, Arong Jung, Ryuichi Nakajima, Yoon-Kyu Song, Bradley J. Baker

**Affiliations:** 1 The Center for Functional Connectomics, Korea Institute of Science and Technology, Seoul, South Korea; 2 Department of Transdisciplinary Studies, Graduate School of Convergence Science and Technology, Seoul National University, Suwon, South Korea; 3 Department of Psychology, Korea University, Seoul, South Korea; 4 College of Life Sciences and Biotechnology, Korea University, Seoul, South Korea; 5 Advanced Institutes of Convergence Technology, Suwon, South Korea; 6 Division of Bio-Medical Science and Technology, KIST School, Korea University of Science and Technology (UST), Seoul, 02792 Republic of Korea

## Abstract

An improved genetically encoded voltage indicator (GEVI) was achieved by altering the charge composition of the region linking the voltage-sensing domain of the GEVI to a pH-sensitive fluorescent protein. Negatively charged linker segments reduced the voltage-dependent optical signal while positively charged linkers increased the signal size. Arginine scanning mutagenesis of the linker region improved the signal size of the GEVI, Bongwoori, yielding fluorescent signals as high as 20% ΔF/F during the firing of action potentials. The speed of this new sensor was also capable of optically resolving action potentials firing at 65 Hz. This large signal size enabled individual pixels to become surrogate electrodes. Plotting the highest correlated pixels based only on fluorescence changes reproduced the image of the neuron exhibiting activity. Furthermore, the use of a pH-sensitive fluorescent protein facilitated the detection of the acidification of the neuron during the firing of action potentials.

## Introduction

Effective imaging of neuronal activity by Genetically Encoded Voltage Indicators (GEVIs) depends on the combination of the magnitude of the voltage-induced optical response, the speed of the fluorescence change, the voltage range that elicits an optical signal, and efficient plasma membrane expression (limited internal expression). These characteristics ultimately determine the Signal-to-Noise Ratio (SNR) which is critical for *in vivo* measurements. Improvements in any of these features can increase the SNR and offset deficiencies in the other characteristics. For instance, ArcLight is a GEVI that has a slow response time but gives one of the largest signal sizes for 100 mV depolarization of the plasma membrane^1, 2^. ArcLight also has a very broad voltage range enabling the detection of all types of neuronal activity ranging from inhibitory activity, to synaptic sub-threshold depolarizations, to action potentials. However, this large voltage range in combination with its slow speed makes it difficult for ArcLight to differentiate synaptic activity from action potentials when imaging populations of cells^3^. Bongwoori is another GEVI that is an ArcLight derivative exhibiting improved speed and a shifted voltage-response to more positive potentials which enabled the optical resolution of action potentials firing at 60 Hz^4^. While an *in vivo* signal could be observed when Bongwoori was expressed in zebrafish embryos, the signal size was small complicating the detection of neuronal activity^5^. In this report, we describe an improved version of Bongwoori yielding optical signals as high as 20% ΔF/F during the firing of action potentials.

**Figure 1:**
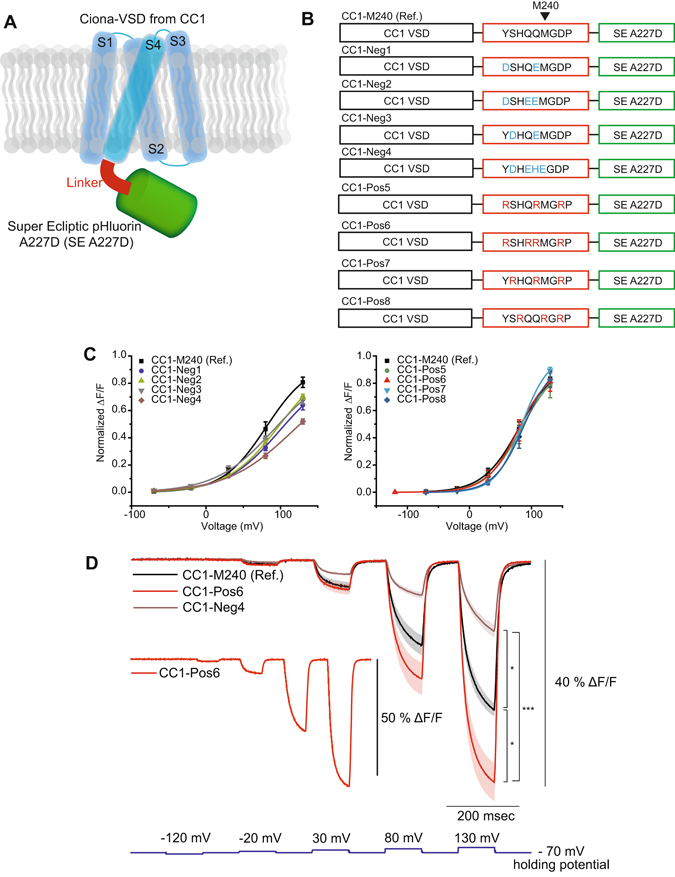
Altering the charge composition of the linker domain affected the size of the voltage-dependent optical signal. (**A**) Schematic of the GEVI. The voltage-sensing domain is in blue. The linker segment is in red, and the FP is in green. (**B**) Composition of the negatively and positively charged linker constructs. The VSD is from CC1, a *Ciona intestinalis* derived GEVI with V_1/2_ near +80 mV. Introduction of negatively charged amino acids in the linker are shown in blue and positively charged ones in the linker are shown in red. (**C**) The Boltzmann fit of normalized optical signal (zero – minimum, one, maximum) in response to voltage. Comparison between CC1-M240 and the negative linker variants (left) and the positive variants (right). (**D**) The averages of CC1-M240, CC1-Pos6 and CC1-Neg4 optical traces responding to stepped voltage pulses. Inset shows a fluorescence trace from an HEK 293 cell expressing CC1-Pos6 with more than 50% signal size during a 200 mV voltage pulse. ‘Ref.’ indicates ‘Reference’. The shaded area denotes standard error of the mean. The number of cells averaged for each construct; CC1-M240: 4, CC1-Neg1: 3, CC1-Neg2: 4, CC1-Neg3: 5, CC1-Neg4: 4, CC1-Pos5: 4, CC1-Pos6: 6, CC1-Pos7: 6, CC1-Pos8: 4. Asterisks in D indicate statistically significant differences between means compared (*p < 0.05 and ***p < 0.001).

Bongwoori and ArcLight consist of a Voltage-Sensing Domain (VSD) fused to a fluorescent protein (FP) via a short linker sequence (Fig. 1). The VSD is a functionally independent structure consisting of four transmembrane segments designated S1-S4 that changes conformation during alterations in membrane potential^6–13^. ArcLight is a fusion of the VSD from a voltage-sensing phosphatase to a pH sensitive Fluorescent Protein (FP) with a unique, external, negatively charged amino acid^1, 14^. A recent report investigating the mechanism mediating this voltage-induced fluorescence change implicated an intermolecular association via the dimerization of the FP^15^. Mutations to the FP that diminish the affinity for dimerization reduced the voltage-dependent optical signal by over 70%. That result suggested the hypothesis that the movement of S4 in response to changes in membrane potential drags the outer negative charge of the FP along the β-barrels of the dimerized FPs transiently affecting the fluorescence of the GEVI.

With S4 movement mediating an optical signal, the amino acids that link the FP to the VSD domain may play an important role in coupling the movement of the VSD domain to changes in fluorescence. Indeed, multiple reports have demonstrated that linker length^1, 4, 14, 16^ and the amino acid composition of the linker^17^ can alter the size, speed and voltage range of the fluorescence change. In this report, we examined the effects of charged residues in the linker domain. The introduction of positively charged arginines into the linker resulted in a probe that gave a large signal of over 50% ΔF/F per 200 mV depolarization. While a 200 mV depolarization is not physiologically relevant, this extreme range enables the observation of the total extent of the optical signal which may be ‘tuned’ to more useful voltages upon further development. Charge alone was not sufficient for this improvement. Replacement of arginine with lysine residues decreased the signal size suggesting that the structure of the linker domain was an important factor in optimizing the coupling of the movement of S4 to a change in fluorescence.

Attempts to focus the optical signal to physiologically relevant potentials resulted in several new probes exhibiting altered voltage-sensing ranges. Fusing these new linker domains to different VSDs that shift the voltage-sensitivity range yielded probes with similar kinetics and signal sizes, but differed in their ability to resolve action potentials. One novel GEVI, Bongwoori-Pos6, exhibited a V_1/2_ (the membrane potential at half the maximal fluorescence change) near −30 mV. Another new GEVI, Bongwoori-R3, had a V_1/2_ close to zero mV. As a result, Bongwoori-R3 was able to resolve action potentials firing at a frequency of 65 Hz and gave signals over 15% ΔF/F per action potential. The increased size of the optical signal enabled the optical reconstruction of a neuron expressing Bongwoori-R3 solely by plotting the pixels with highly correlated fluorescence intensities during neuronal activity.

## Materials and Methods

### Plasmid DNA construction

Conventional one-step and two-step overlap extension PCRs were used to generate linker variant constructs. CC1-Neg1 was derived from CC1^4^. The initial PCR used primers RK024 (forward; 5′- aagctggctagcaccATGGAGGGATTCGACGGTTCAG-3′) and neg1-B (reverse; 5′-GGGATCCCCCATC TCTTGGTGGGAGTCAAATATTCTTGC-3′) to amplify the voltage-sensing domain of CC1. The FP part for super ecliptic pHluorin A227D (SE A227D) was amplified via PCR using Bongwoori as template and neg1-A (forward; 5′-GAATATTTGACTCCCACCAAGAGATGGGGGATCCCATGA G-3′) and RK027 (reverse; 5′- ttttctcgAGTTCTAGATCATTTGTATAGTTCATCC-3′). The overlapped linker regions from both PCR products annealed to each other in a second PCR reaction and then amplified by the flanking primers, RK024 and RK027. The final PCR product was digested with Nhe1 and Xho1 and then ligated into pcDNA3.1 / Hygro ( + ) backbone vector (Invitrogen, USA). Subsequently, other linker variants (CC1-Neg2, CC1-Neg3, CC1-Neg4, CC1-Pos5, CC1-Pos6, CC1-Pos7, CC1-Pos8 and CC1-M240) were cloned by using CC1-Neg1 as a template DNA. Constructs consisting of a single type of amino acid residue in their linker regions (CC1-9Ds, CC1-9Es, CC1-9Ks, CC1-9Rs, CC1-9Ss, CC1-9Qs and CC1-9As) were mutated from CC1-M240. CC1-Pos6 was used as a template DNA construct for generation of D164N-Pos6 and CC1-Pos6-K gene constructs. For generation of Bongwoori-Pos6, two different primers encoding CC1-Pos6′s linker sequence were used for a PCR reaction onto Bongwoori and for another PCR reaction onto CC1-Pos6. The two resulting PCR products were then annealed together and then amplified by RK024 and RK027. Eight constructs tested for Bongwoori-arginine scanning experiment were mutated from Bongwoori with primers designed to introduce an arginine at each of the 8 different linker position. All the primers were synthesized commercially and the generated DNA constructs were verified by DNA sequencing (both by Cosmogenetech, South Korea). The primer sequences are provided in the supplementary table.

### Cell culture and transfection

HEK 293 cells were cultured in 10% Fetal Bovine Serum (Gibco, USA) supplemented Dulbecco’s Modified Eagle Medium (DMEM; Gibco, USA) at 37 °C and 5% CO_2_. On the day of transfection, HEK 293 cells were suspended with 0.25% trypsin-EDTA solution (Gibco, USA) and plated onto poly-L-lysine (Sigma-Aldrich, USA) coated #0 coverslip (0.08-0.13mm thick and 10 mm diameter; Ted Pella, USA). Then, Lipofectamine 2000 (Invitrogen, USA) was used for transient transfection following the manufacturer’s protocol.

Hippocampal neurons were obtained according to an approved animal experiment protocol by the Institutional Animal Care and Use Committee at KIST (animal protocol 2014-001). Hippocampi of embryonic day 17 C57BL/6 mice (Koatech, South Korea) were dissected for primary neuron culture as previously described with some modifications^18^. The dissected hippocampi were incubated with 0.125% Trypsin-EDTA solution (Gibco, USA) for 20 min in 37 °C water bath. Prior to mechanical trituration, DNase1 (Sigma Aldrich, USA) was added to the tube to minimize clogging of the cells for 30 sec and then washed out with plating medium (PM) composed of 10% FBS and 1% penicillin-streptomycin (Gibco, USA) in DMEM. The dissociated neurons were then plated onto poly-D-lysine (Sigma-Aldrich, USA) coated #0 coverslips at 50,000 cells/mL density. After three hours of incubation in a 5% CO_2_ incubator at 37 °C, PM was gently changed to maintenance medium (MM) that was supplemented with 2% B-27 supplement (Gibco, USA), 1% penicillin-streptomycin and 0.25% Glutamax (Gibco, USA) in Neurobasal medium (Gibco, USA). Fresh MM was used to exchange half of the media in each well on every third day throughout the culture. Transient transfection for cultured mouse hippocampal neurons were typically done on days *in vitro* (DIV) 5–7 using Lipofectamine 2000 according to the manufacturer’s protocol and then experimented on DIV 8–12.

### Electrophysiology

Coverslips with transiently transfected cells were inserted into a patch chamber (Warner instruments, USA) with its bottom side covered with a #0 thickness cover glass for simultaneous fluorescence imaging. The chamber was kept at 34 °C throughout the experiment and perfused with bath solution (150 mM NaCl, 4 mM KCl, 1 mM MgCl_2_, 2 mM CaCl_2_, 5 mM D-glucose and 5 mM HEPES, pH = 7.4). Filamented glass capillary tubes (1.5mm/0.84mm; World Precision Instruments, USA) were pulled by a micropipette puller (Sutter, USA) prior to each experiment to pipette resistances of 3–5 MΩ for HEK 293 cells and 3–6 MΩ for cultured primary neurons. The pipettes were filled with intracellular solution (120mM K-aspartate, 4 mM NaCl, 4 mM MgCl_2_, 1 mM CaCl_2_, 10 mM EGTA, 3 mM Na_2_ATP and 5 mM HEPES, pH = 7.2) and held by a pipette holder (HEKA, Germany) mounted on a micromanipulator (Scientifica, UK). Whole cell voltage clamp and current clamp recordings of transfected cells were conducted using a patch clamp amplifier (HEKA, Germany). A holding potential of −70 mV was used for all recordings including neuronal recording with cultured neurons until it was switched to current clamp mode. The internal solution with high buffering capacity (100 mM HEPES, pH = 7.2) was prepared as previously described (Kang & Baker 2016).

### Fluorescence microscopy

An inverted microscope (IX71; Olympus, Japan) equipped with a 60X oil-immersion lens with 1.35-numerical aperture (NA) was used for epifluorescence imaging. The light source was a 75 W Xenon arc lamp (Osram, Germany) placed in a lamp housing (Cairn, UK). A filter cube consisting of an excitation filter (FF02-472/30), a dichroic mirror (FF495-Di03) and an emission filter (FF01-497/LP) (all by Semrock, USA) was used for excitation and acquisition of emitted green fluorescence. Two cameras were mounted on the microscope through a dual port camera adapter (Olympus, Japan). A slow speed color charge-coupled-device (CCD) camera (Hitachi, Japan) was used to aid locating cells and pipettes during patch clamp experiments. Fluorescence changes of the voltage indicators were typically recorded at 1 kHz frame rate by a high-speed CCD camera (RedShirtImaging, USA). All the relevant optical devices were placed on a vibration isolation platform (Kinetic systems, USA) to avoid any vibrational noise during patch clamp fluorometry experiments.

Two-photon imaging of HEK 293 cells was conducted by using FV1000 MPE multiphoton laser scanning microscope system (Olympus, Japan). Femtosecond-pulse laser illumination (Mai Tai DeepSee, Spectra-Physics, USA) at 940 nm wavelength was provided through a 25 × 1.05 NA water immersion objective lens (Olympus, Japan). The laser power measured at the end of the objective lens was 5.2 mW. Acquisition rate of the recording was 5.3 Hz on 128 × 128 pixels. Emitted light from fluorescent proteins first passed a dichroic mirror (RDM690) and then a filter cube equipped with a beam splitter (DM 570) and a band pass filter with a 495–540 nm bandwidth (All by Olympus, Japan). The optical signals were collected by a multi alkali photomultiplier tube (Olympus, Japan). Fluoview FV1000 software (Olympus, Japan) was used for controlling the microscope system and acquisition of resulting images. Patch clamp recording conditions such as temperature, internal solution and bath solution were the same as described earlier for single-photon fluorescence microscopy imaging. The electrical signals from the whole cell voltage clamp recording were amplified and digitized by Multi-clamp 700B amplifier and Digidata 1440 A digitizer (both by Molecular devices, USA).

Photobleaching of HEK 293 cells expressing ArcLight A242, Bongwoori or Bongwoori-R3 was conducted using a 75 W Xenon arc-lamp (Osram, Germany) at a light intensity that was used for all the other experiments in this work except for the two-photon imaging. The excitation light intensity measured at the specimen plane was 1 mW/mm^2^. Each trial (40 s light-on / 20 s light-off) was repeated to bleach a cell in the patching chamber that was maintained at 34 °C and with continuous perfusion of normal patch clamp bath solution (see above). Images were taken by a high-speed CCD camera (RedShirtImaging, USA) at 40 Hz during the 40 s light-on period. Fluorescence intensities were spatially averaged from the whole cell area and plotted as a function of emitted fluorescence versus cumulative excitation time. The plotted values were fitted onto an exponential decay function in OriginPro 8.6 (OriginLab, USA). Time constants from the exponential fits were calculated to quantify the photobleaching rate of each construct.

### Data acquisition and analysis

Resulting images from patch clamp fluorometry were acquired and analyzed for initial parameters such as fluorescence change [ΔF = Fx−F0] or fractional fluorescence change values [ΔF/F = ((Fx−F0)/F0) * 100] by Neuroplex (RedShirtImaging, USA) and Microsoft Excel (Microsoft, USA). The acquired data from whole cell voltage clamp experiments of HEK 293 cells were averaged for 16 trials (technical replication) unless otherwise noted. The number of recorded cells in this work is biological replicates and the number of trials averaged for each HEK 293 cell should be interpreted as technical replication. Data were collected from recorded cells that did not lose their seals during the whole cell voltage clamp recording. ΔF/F values for the tested voltage pulses were plotted in OriginPro 2016 (OriginLab, USA) and fitted to a Boltzmann function (signal size was normalized from zero, minimum, to one, maximum) to acquire voltage sensitivities as previously described^4^. A ΔF/F trace versus time graph for each cell was also fitted for either double or single exponential decay functions in OriginPro 2016 as described previously^4^.

Neuronal recordings were imaged in single trials under whole cell current clamp mode. Comparison of five GEVIs was done by analyzing three representative cells for each probe showing good membrane expression, signal size, and firing rates of 15–25 Hz during 200 msec current pulse. The brightness at resting state, ΔF, ΔF/F and optical signal size ratio of a spike (from threshold to the peak) to total (from resting state to the peak) were analyzed by using Neuroplex and Excel. The correlation coefficients between ΔF/F values of every pixel and electrically measured voltage signals were then calculated for the 15 recordings, using custom codes in MATLAB (Mathworks, USA). The resulting matrix of R-values for each recording was then sorted to find the pixel with maximum coefficient for subsequent single pixel imaging analysis. Signal-to-Noise Ratios (SNRs) were calculated by dividing ΔF/F at the time point of the AP peak by noise value, which was determined by the standard deviation of the first 100 frames before any electrical stimulation. SNR of single pixel imaging results were calculated from traces low-pass filtered with Gaussian filter with a cut-off frequency of 100 Hz.

The correlation between ΔF/F values of each pixel to all the other pixels was also calculated and resulted in a correlation matrix of the pixels’ traces at different times of interest. The matrix was sorted to extract the best-correlated pixel pairs that in turn were identified on a reconstructed mask of the image (1 if identified as belonging to one of the nth best-correlated pixel pair, 0 otherwise). This procedure was used during three different times of interest, for the subthreshold, the resting state, and action potentials, each consisting of 150 frames.

### Statistics

Error bars in column graphs and the shaded area in the averaged optical traces represent standard error of the mean (SEM). Means are presented as mean ± SEM. The sample sizes were not determined by a statistical method but they are similar to those used by others in the field. Prior to statistical analyses to compare means from different groups, the Shapiro-Wilk test was used to determine normality of data. The means from two different groups following normal distribution were compared by a two tailed Student’s t-test. For comparison of means from three or more groups, one-way analysis of variance (ANOVA) followed by the post-hoc Tukey test was performed to first confirm statistical difference between means and then to compare the means to each other. For groups that did not follow normal distribution, the Kruskal-Wallis ANOVA followed by the Dunn’s multiple comparisons test were used. The significance level of 0.05 was applied to all comparisons. All statistical analyses were performed by using OriginPro 2016 and Prism 7 (GraphPad, USA).

### Baseline change analysis of neurons

The relationship between the number of evoked action potentials and the amplitude of baseline change was analyzed with cultured neurons expressing Bongwoori-R3. Both pipette solutions with normal and high buffering capacities were used for 200 msec pulse and 800 msec pulse on 14 cells. Then baseline ΔF/F differences before and after injected current pulses were calculated but any recording that underwent more than 3 mV drift in voltage was excluded from analysis to avoid including optical signal change responding to real membrane potential change.

## Results

### Positively charged linker regions improved the magnitude of the voltage-induced optical signal while negatively charged linker regions reduced the signal

The length and amino acid composition of the linker between S4 of the voltage-sensing domain and the FP affect the optical characteristics of GEVIs^1, 4, 14, 16, 17^. One potential mechanism to explain these results is that the amino acids linking the FP to the VSD are capable of responding to changes in the voltage field since they are close to the plasma membrane. To test this hypothesis, a panel of GEVIs consisting of the VSD from CC1-M240 with varying positively and negatively charged linker segments was tested in HEK 293 cells (Fig. 1). The GEVI, CC1-M240, has a V_1/2_ near + 80 mV (Table 1). Since the movement of the S4 transmembrane segment results in an optical signal^15, 19^, we employed a VSD that requires a strong depolarization of the plasma membrane of around 100 mV to elicit an optical signal. The use of the CC1-M240 VSD thereby limits the movement of S4 during weaker depolarizations of the plasma membrane potentially enhancing the detection of linker-induced fluorescence changes in response to voltage.

**Table 1:** The Boltzmann fit results of GEVIs tested in HEK 293 cells.

Constructs	V_1/2_ (mV)	dx (slope)
CC1-M240 (Ref.)	81 ± 4	29 ± 1
CC1-Neg1	98 ± 13	36 ± 4
CC1-Neg2	100 ± 7	36 ± 3
CC1-Neg3	92 ± 16	39 ± 6
CC1-Neg4	116 ± 30	44 ± 9
CC1-Pos5	84 ± 1	29 ± 1
CC1-Pos6	78 ± 7	25 ± 2
CC1-Pos7	85 ± 1	21 ± 1
CC1-Pos8	88 ± 1	24 ± 1
D164N-Pos6	37 ± 6	23 ± 3
Bongwoori-Pos6	−28 ± 3	27 ± 1
Bongwoori-R3	−3 ± 1	29 ± 1
Bongwoori	6 ± 1	25 ± 1

V_1/2_ is the membrane potential at half the maximal fluorescence change. Values were shown as mean ± SEM (standard error of the mean). The number of cells averaged; CC1-M240: 4, CC1-Neg1: 3, CC1-Neg2: 4, CC1-Neg3: 5, CC1-Neg4: 4, CC1-Pos5: 4, CC1-Pos6: 6, CC1-Pos7: 6, CC1-Pos8: 4, D164N-Pos6: 6, Bongwoori-Pos6: 4, Bongwoori-R3: 5, Bongwoori: 4.

Four GEVIs with negatively charged linkers (CC1-Neg 1–4) and four GEVIs with positively charged linker segments (CC1-Pos 5–8) varying in number and position of the charged amino acids were expressed in HEK 293 cells and subjected to whole cell voltage clamp. As can be seen in Fig. 1C, none of these GEVIs were able to give an optical signal below membrane potentials of +30 mV. The negatively charged linkers reduced the size of the optical signal. While the GEVIs with positively charged linker segments did not significantly affect the voltage sensitivity of the probe, the signal strength was substantially improved with the best trial from CC1-Pos6 giving a 55% ΔF/F for a 200 mV depolarization step (Fig. 1D).

### The linker composition improves the optical signal by orientation of the FP

**Figure 2:**
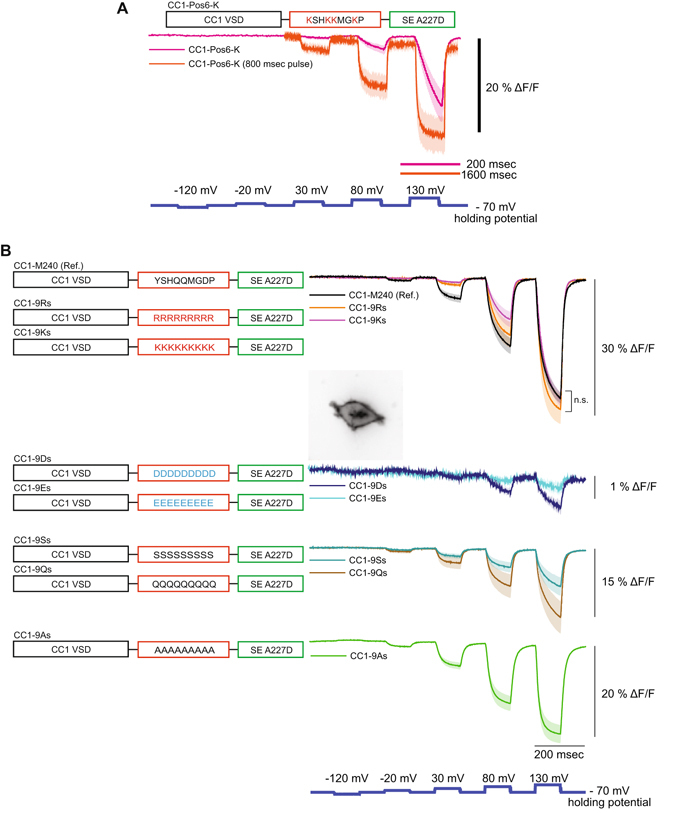
Effects of altered linker charge composition. (**A**) The lysine version of CC1-Pos6, CC1-Pos6-K, showed a reduced signal size and slower response. (**B**) Linker variants consisting of a single amino acid type. Positively charged linkers had the least effect on signal size for a 200 mV depolarization and did not show statistical difference compared to CC1-M240 (n.s.: not significant). Negative amino acid linkers reduced the signal size nearly 15-fold. Inset shows an HEK 293 cell expressing CC1-9Ds. Polar linkers consisting of serine or glutamine reduced the signal by roughly 50%. A linker consisting entirely of alanines reduced the signal size by about a third. The shaded area denotes standard error of the mean. The number of cells averaged for each construct; CC1-M240: 4, CC1-Pos6-K: 4, CC1-Pos6-K (800 msec pulse): 4, CC1-9Rs: 4, CC1-9Ks: 4, CC1-9Ds: 3, CC1-9Es: 3, CC1-9Ss: 4, CC1-9Qs: 4, CC1-9As: 5. Scale bar = 20 µm. In Fig. 2B, no significant difference (n.s.) was found between the three groups.

To further examine the role of positive charges in the linker segment, the arginines in CC1-Pos6 were changed to lysines (CC1-Pos6-K). As can be seen in Fig. 2A, the signal size for CC1-Pos6-K was reduced by nearly half. The speed of the response time was also much slower for CC1-Pos6-K. These results implied that charge alone was not sufficient to improve the optical signal of CC1-Pos6. The overall structure of the linker domain was important. GEVIs with linkers consisting entirely of arginines or lysines supported that conclusion. When the linker consisted of nine arginines or nine lysines, there was no statistical difference in the speed or size of the voltage-dependent optical signal when compared to the original CC1-M240 construct (Fig. 2B). Increasing the number of positive charges did not improve the signal.

Two probes consisting entirely of negatively charged amino acid residues (D or E) in their linkers were also tested (Fig. 2B). Surprisingly, voltage-dependent optical signals for these probes nearly vanished despite having good membrane expression (Fig. 2B inset). In addition to CC1-9Ds and CC1-9Es, several constructs with other types of amino acid residues were tested. Regardless if the linker contained alanine residues or polar residues (either nine serines or nine glutamines), the signal size was not as large as CC1-Pos6. CC1-Pos6 was therefore chosen for further development.

### The Pos6 linker can shift the voltage response of a GEVI to more negative potentials or more positive potentials depending on the VSD

**Figure 3:**
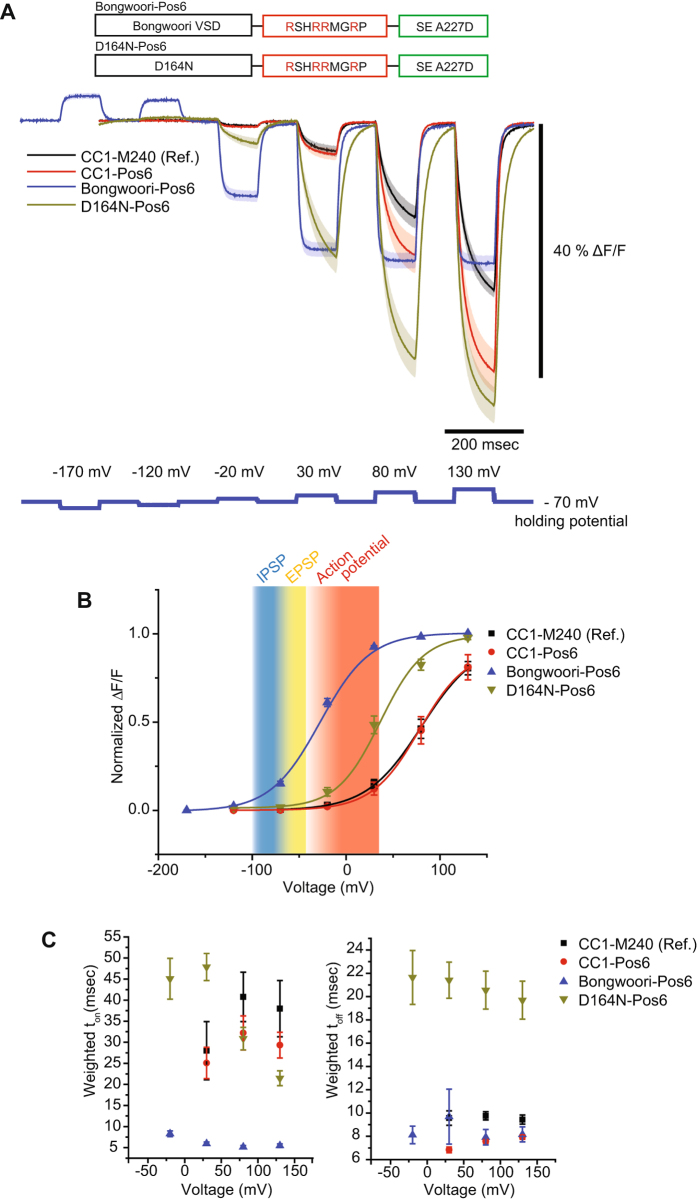
Adjusting the voltage sensitivity to a physiologically relevant voltage range. (**A**) Schematic showing the different VSD domains fused to the Pos6 linker and their responsive optical signals to stepped voltage pulses in HEK 293 cells. The traces from CC1-M240 and CC1-Pos6 are also shown for comparison. (**B**) The Boltzmann fit of the normalized data in (**A**) The background coding depicts the different types of neuronal activity with inhibitory post-synaptic potentials (IPSPs) shaded blue, excitatory post-synaptic potentials (EPSPs) shaded yellow, and action potentials shaded red. (**C**) The weighted time constants showing speed of the optical response as a function of voltage. For Bongwoori-Pos6 traces in (**A**), 12 trials were averaged. The shaded area and error bars denote standard error of the mean. The number of cells averaged for each construct; CC1-M240: 4, CC1-Pos6: 6, Bongwoori-Pos6: 4 and D164N-Pos6: 6.

As reviewed in Nakajima *et al*.^3^, the voltage range of a GEVI is an important characteristic in determining the types of neuronal activity to be optically resolved. Mutagenesis of the VSD can change the voltage range and speed of the optical signal^4, 20, 21^. To ‘tune’ CC1-Pos6 to physiologically relevant potentials, two sets of mutations were introduced into the VSD. The first set of mutations consisted of the three changes used to produce the GEVI, Bongwoori^4^, one mutation in the S2 transmembrane segment (A154D) and two mutations in the S4 transmembrane segment (R217Q/R229I). Combining the Bongwoori VSD with the Pos6 linker, designated as Bongwoori-Pos6, resulted in a GEVI with faster kinetics having an on tau of 6 ± 1 msec and an off tau of 8 ± 1 msec (Fig. 3 and Table 1). The signal size also improved to nearly 20% ΔF/F per 100 mV depolarization of the plasma membrane. However, the Bongwoori-Pos6 linker shifted the voltage dependence of the GEVI to more negative potentials than expected. Bongwoori had a V_1/2_ of 6 mV which is fairly consistent with the V_1/2_ of −3 mV reported previously^4^. Bongwoori-Pos6, in contrast, had a V_1/2_ of −28 mV.

The second attempt to ‘tune’ the voltage response of CC1-Pos6 consisted of a single mutation to the S2 domain (D164N). Piao *et al*.^4^ reported that the VSD of the *Ciona* phosphatase containing the D164N mutation had a V_1/2_ of 6 mV. Fusing the Pos6 linker to the D164N VSD shifted the voltage response to more positive potentials (Fig. 3 and Table 1). D164N-Pos6 had a V_1/2_ of +37 mV. While the Pos6 linker shifted the optical response of Bongwoori to more negative potentials, the Pos6 linker fused to the D164N VSD shifted the optical response to more positive potentials. The linker length of D164N-Pos 6 is shorter than the construct reported in Piao *et al*.^4^ which could affect the voltage-sensitivity of the probe as well. D164N-Pos6 also exhibited much slower kinetics with a weighted tau on of 48 ± 3 msec and a tau off best fit by a single exponential at 21 ± 2 msec (Table 2).

**Table 2:** Kinetics of GEVIs tested in HEK 293 cells.

Constructs	State	Weighted τ (msec)	Fast τ (msec)	Slow τ (msec)	% fast
CC1-M240	On	28 ± 7	15 ± 3	56 ± 16	72 ± 16
	Off	10 ± 1	8 ± 1	24 ± 1	83 ± 7
CC1-Pos6	On	25 ± 4	12 ± 2	60 ± 6	73 ± 8
	Off	7 ± 1	7 ± 1	—	100
D164N-Pos6	On	48 ± 3	17 ± 8	54 ± 2	32 ± 14
	Off	21 ± 2	21 ± 2	—	100
Bongwoori-Pos6	On	6 ± 1	6 ± 1	—	100
	Off	10 ± 2	8 ± 1	80*	97 ± 3
Bongwoori-R3	On	11 ± 1	7 ± 1	45 ± 1	90 ± 1
	Off	10 ± 2	6 ± 1	46 ± 6	91 ± 1
Bongwoori	On	17 ± 1	9 ± 1	40 ± 4	76 ± 2
	Off	14 ± 1	8 ± 1	52 ± 9	86 ± 1

Values are shown as the mean ± SEM (standard error of the mean). The number of cells analyzed; CC1-M240: 4, CC1-Pos6: 6, D164N-Pos6: 6, Bongwoori-Pos6: 4, Bongwoori-R3: 5, Bongwoori: 4. *Only one cell showed slow component for tau off at a 100 mV pulse.

Both attempts to tune CC1-Pos6 to physiologically relevant membrane potentials successfully shifted the voltage dependent optical response to more negative potentials. However, the Bongwoori-Pos6 construct shifted the V_1/2_ too far towards negative potentials while the D164N-Pos6 did not shift the voltage response enough. These results demonstrated the need to empirically determine the optimal amino acid composition of the linker region.

### Arginine scanning of the linker domain improves the signal strength of Bongwoori while maintaining the voltage sensitivity of the optical signal

**Figure 4:**
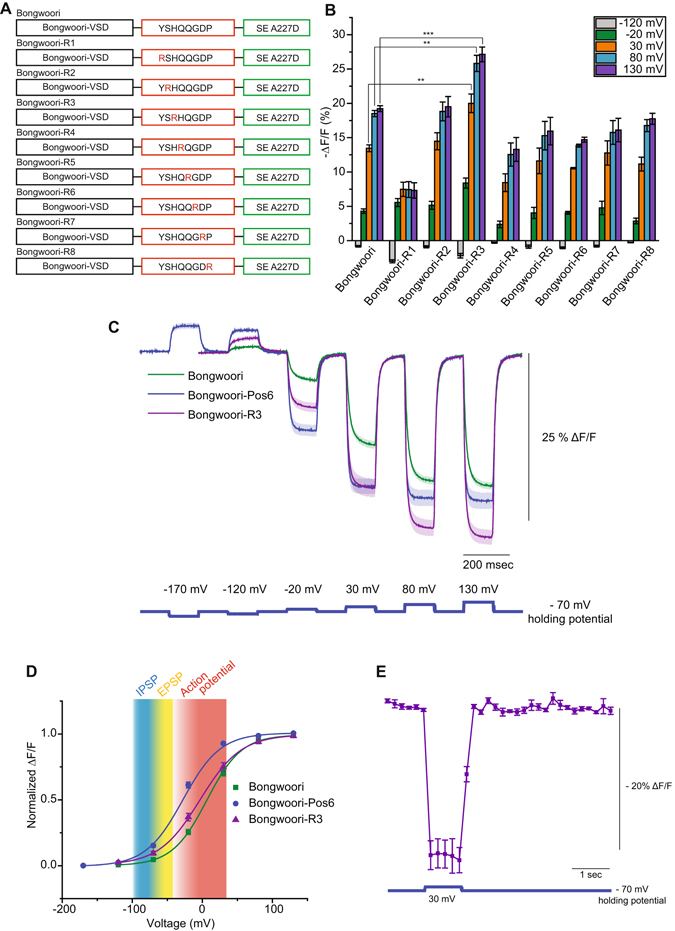
Arginine scanning of the linker domain. (**A**) An arginine residue (red) was introduced at every position between the S4 transmembrane segment and the FP. (**B**) Bar graph of the optical responses for the constructs in A at 5 different membrane potentials. (**C**) Comparison of the optical response of Bongwoori-R3 to Bongwoori and Bongwoori-Pos6. (**D**) The Boltzmann fit of the normalized data in (**C**) Shading is as Fig. 3. The shaded area and error bars denote standard error of the mean. (**E**) Two-photon voltage imaging of Bongwoori-R3 expressing HEK 293 cells responding to a 100 mV depolarizing voltage pulse. The number of HEK 293 cells averaged for each construct; Bongwoori: 4, Bongwoori-R1: 4, Bongwoori-R2: 5, Bongwoori-R3: 5, Bongwoori-R4: 4, Bongwoori-R5: 4, Bongwoori-R6: 4, Bongwoori-R7: 4, Bongwoori-R8: 4 and Bongwoori-Pos6: 4. For two-photon voltage imaging result in (**E**), eight trials were averaged for each cell and the number of cells analyzed was 3. Asterisks in (**B**) indicate statistically significant differences between means compared (**p < 0.01 and ***p < 0.001).

The unpredictable results of combining different linker domain compositions with altered VSDs required a change in strategy for improving the signal strength of Bongwoori. The CC1-Pos6 construct suggested that the position of arginines in the linker domain was important in improving the optical signal. However, the V_1/2_ of Bongwoori-Pos6 and D164N-Pos6 suggested that the optimal positions of arginines in the linker for Bongwoori were probably different from that for CC1-M240. We therefore conducted an arginine scan of the linker domain for Bongwoori. Using the Bongwoori VSD, an arginine was tested at every position in the linker resulting in eight new constructs whose signal strengths can be seen in Fig. 4B. Only the H237R mutation in the linker domain resulted in a GEVI with an improved signal strength. Since this construct mutated the third position of the linker, it was designated Bongwoori-R3.

Bongwoori-R3 exhibits similar kinetic responses to the original Bongwoori construct and a bleaching rate similar to that of ArcLight^22^ (Supplementary Figure 1). The fast component of the on tau, which constitutes 90% of the amplitude of the response, is 6–8 msec (Fig. 4C and Table 2). The fast component of the off tau which also accounts for 90% of the total amplitude is 5–7 msec. A comparison of optical responses for Bongwoori, Bongwoori-Pos6, and Bongwoori-R3 is shown in Fig. 4C. Both Bongwoori-Pos6 and Bongwoori-R3 have improved signal strengths for a 100 mV depolarization of the plasma membrane compared to Bongwoori. The optical signal for Bongwoori-R3 continues to increase for stronger depolarizations of the plasma membrane due to its V_1/2_ being close to zero mV (Fig. 4D).

Bongwoori-R3 also gives a comparable voltage-induced optical signal under 2-photon microscopy (Fig. 4E). Historically, the frame rates of 2-photon microscopy have favored calcium imaging over that of voltage. However, as the speed of 2-photon microscopy improves^23^, it is important to develop GEVIs that are capable of both one-photon and 2-photon imaging. Bongwoori-R3 gives a 21% ΔF/F per 100 mV depolarization of the plasma membrane suggesting that Bongwoori-R3 may be conducive for 2-photon imaging.

### The improved signal size combined with the V_1/2_ near zero mV resulted in improved optical detection of action potentials

**Figure 5:**
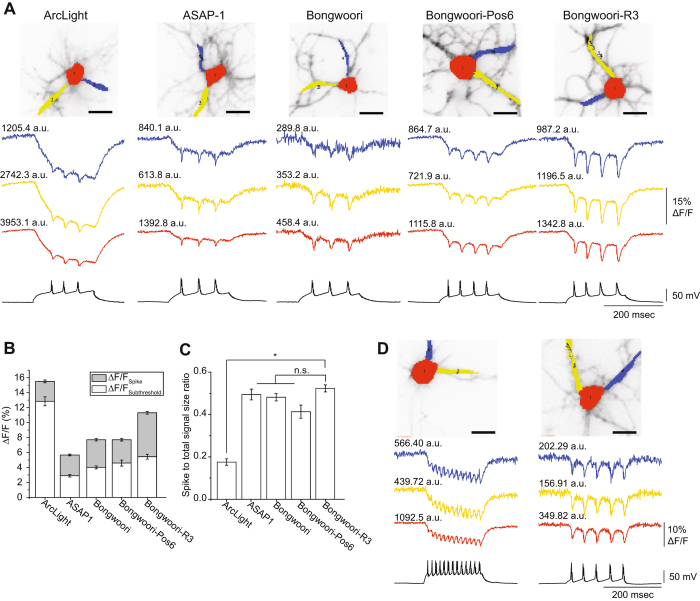
Comparison of different GEVIs’ ability to resolve action potentials. (**A**) Optical traces from three different regions (soma – red, processes – blue and yellow) of cultured mouse hippocampal neurons expressing either ArcLight, ASAP-1, Bongwoori, Bongwoori-Pos6, or Bongwoori-R3 under whole cell current clamp mode. Numbers above each trace refer to the fluorescence intensities (a.u., arbitrary unit) of the chosen pixels. (**B**) Comparison of the signal size of different GEVIs averaged from pixels corresponding to the soma. The bottom white area of the bar graph depicts the size of the optical response to subthreshold depolarization of the plasma membrane. The gray shaded region corresponds to the size of the optical signal during the spike of the action potential. (**C**) Spike to total signal size ratio of the optical signal averaged in (**B**) (n.s., not significant). (**D**) Two hippocampal neurons expressing Bongwoori-R3. The cell on the left is firing at 65 Hz. The cell on the right exhibits lower fluorescence but still yields a robust optical signal in response to action potentials. Error bars and shaded area are standard error of the mean. The number of action potentials (technical replication) averaged for each cell analyzed in Fig. 5B and C are as follows; 3, 5 and 4 for ArcLight, 5, 3 and 4 for ASAP1, 3, 3 and 5 for Bongwoori, 4, 4 and 3 for Bongwoori-Pos6 and 4, 4 and 4 for Bongwoori-R3. The p-values for Fig. 5C were 0.0349 for ArcLight and Bongwoori-R3, >0.9999 for ASAP-1 and Bongwoori-R3, >0.9999 for Bongwoori and Bongwoori-R3, and 0.8284 for Bongwoori-Pos6 and Bongwoori-R3. The asterisk in C indicates a statistically significant difference between the means compared (*p < 0.05). Scale bar = 20 µm.

Since Bongwoori-R3 gave a larger signal than Bongwoori yet had similar kinetics, we felt this would be a better sensor for detecting action potentials. To determine the usefulness of Bongwoori-R3 for monitoring neuronal activity, we compared the optical signals resulting from induced action potentials in hippocampal neurons expressing Bongwoori-R3 to other GEVIs composed of VSDs derived from voltage-sensing phosphatase proteins including ArcLight^1^, ASAP-1^24^, and Bongwoori. Figure 5 compares the optical signal of these GEVIs in response to action potentials from three different regions of the cell, the soma (red trace) and two separate processes (dark blue and yellow traces). While all constructs were able to resolve action potentials optically regardless of which section of the cell was examined, Bongwoori-R3 consistently gave the most robust optical signals in response to action potentials. Bongwoori-R3 had a SNR comparable to ArcLight and twice that of ASAP-1 (Table 3). ASAP-2f was not tested since its physical parameters are virtually the same as ASAP-1 with the exception of ASAP-2f giving a larger signal when hyperpolarizing the membrane potential from −70mV to −120 mV^25^.

**Table 3:** Characteristics of GEVIs resolving evoked action potentials from cultured mouse hippocampal neurons.

Soma (Spatially averaged)	ArcLight A242	ASAP1	Bongwoori	Bongwoori-Pos6	Bongwoori-R3
Brightness (a.u.)	1923 ± 350	1162 ± 57	558 ± 41	1034 ± 74	1055 ± 91
ΔF/F - total (%)	15.5 ± 0.5	5.7 ± 0.1	7.7 ± 0.3	7.7 ± 0.4	11.3 ± 0.4
ΔF/F - spike (%)	2.7 ± 0.2	2.8 ± 0.1	3.7 ± 0.2	3.1 ± 0.2	5.9 ± 0.2
Spike to total signal size ratio	0.18 ± 0.02	0.49 ± 0.03	0.48 ± 0.02	0.41 ± 0.03	0.52 ± 0.02
SNR	68 ± 7	26 ± 1	19 ± 1	33 ± 3	52 ± 4
Action potential amplitude (mV)	86 ± 1	85 ± 3	96 ± 2	85 ± 4	80 ± 2

All analyzed data were acquired by spatially averaging pixels corresponding to the soma. Values were shown as mean ± SEM (standard error of the mean). The number of action potentials analyzed; ArcLight: 12, ASAP1: 12, Bongwoori: 11, Bongwoori-Pos6: 11, Bongwoori-R3: 12, all from three cells for each GEVI.

Figure 5 also illustrates the effect of the voltage-dependence of the GEVI on the optical signal. With a V_1/2_ near zero mV, Bongwoori-R3 gives a better ‘contrast’ for the spiking activity of the neuron. While Bongwoori-Pos6 has similar kinetics to Bongwoori-R3, the V_1/2_ is shifted towards subthreshold potentials thereby reducing the contrast of the spiking activity (Fig. 5C). Even though Bongwoori-R3 is not as fast as ASAP-1 (taus around 2 msec)^26^, its larger signal size (and decent speed) enable Bongwoori-R3 to better resolve action potentials firing at a frequency as high as 65 Hz (Fig. 5D). Indeed, the CC1-Pos6 and the D164N-Pos6 constructs do not resolve spiking activity in neurons as well as Bongwoori-R3 (Supplementary Figure 2).

### Baseline drift of the fluorescence for Bongwoori when expressed in neurons is due to the acidification of the cell firing action potentials

One drawback in the original publication of Bongwoori was that for neuronal recordings the baseline fluorescence tended to drift. Although the baseline drift did not hinder the resolution of action potentials firing at 60 Hz, we were curious as to why the drift was seen only in neuronal recordings and not when Bongwoori was expressed in HEK cells^4^.

**Figure 6:**
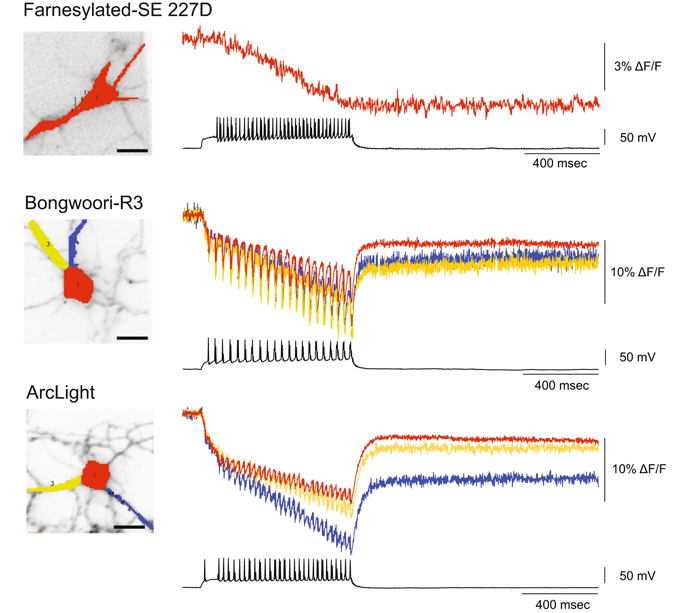
The baseline drift is due to the acidification of the neuron during the firing of action potentials. Cultured hippocampal neurons expressing a farnesylated version of the FP, SE 227D which is pH-sensitive, Bongwoori-R3, or ArcLight. To determine the role of pH induced fluorescent effects, a lipid-anchored version of SE 227D was expressed in hippocampal neurons and subjected to action potentials. The cultured hippocampal neuron expressing Bongwoori-R3 enables the optical resolution of action potentials and allows the comparison of the baseline changes in the soma to the processes. The neuron expressing ArcLight is also able to resolve action potentials and exhibits the baseline drift due to pH. Red trace is from the soma. Blue and yellow traces are from the processes. Scale bar = 20 µm.

Bongwoori, Bongwoori-Pos6, and Bongwoori-R3 all contain an FP sensitive to physiological pH^27, 28^ that has a negative charge on the outside of the β-can structure. We recently reported the effects of pH on the optical signal for another GEVI, Pado, that uses the same FP fused to a VSD from a voltage-gated proton channel^15^. Since the baseline drift for Pado was a response to a change in intracellular pH, we tested whether Bongwoori-R3 was responding to acidification of the neuron during the firing of action potentials. To visualize the effects of pH, neurons were transfected with a farnesylated version of SE 227D^15^ which is sensitive to physiological pH but not voltage (there is no VSD) and subjected to current clamp for induction of action potentials. Figure 6 shows a clear decrease in the fluorescence as a response to the firing of action potentials. This is not bleaching since the baseline stays steady after the current injection has ended. The baseline drift does not inhibit the resolution of action potentials by Bongwoori-R3 as can be seen in Fig. 6. The baseline change was more pronounced in the processes than in the soma (Fig. 6) potentially due to the higher buffering capacity of the soma. Neurons are known to acidify upon the firing of action potentials^29^. In agreement, the amount of the baseline fluorescence change correlated with the number of action potentials fired (Supplementary Figure 3). We normally do not try to image high frequency action potentials in neurons expressing ArcLight since it is a slower probe with an on tau of 10 msec (fast component) and an off tau over 30 msec^1^. However, ArcLight was able to resolve spikes at around 35 Hz though the contrast for the spikes was lower than that seen in Bonwoori-R3 (Fig. 6). ArcLight also exhibits a similar pH-induced baseline effect as seen in Bongwoori.

### Correlating pixels based on fluorescence change enables the optical reconstitution of the cell based on neuronal activity

**Figure 7:**
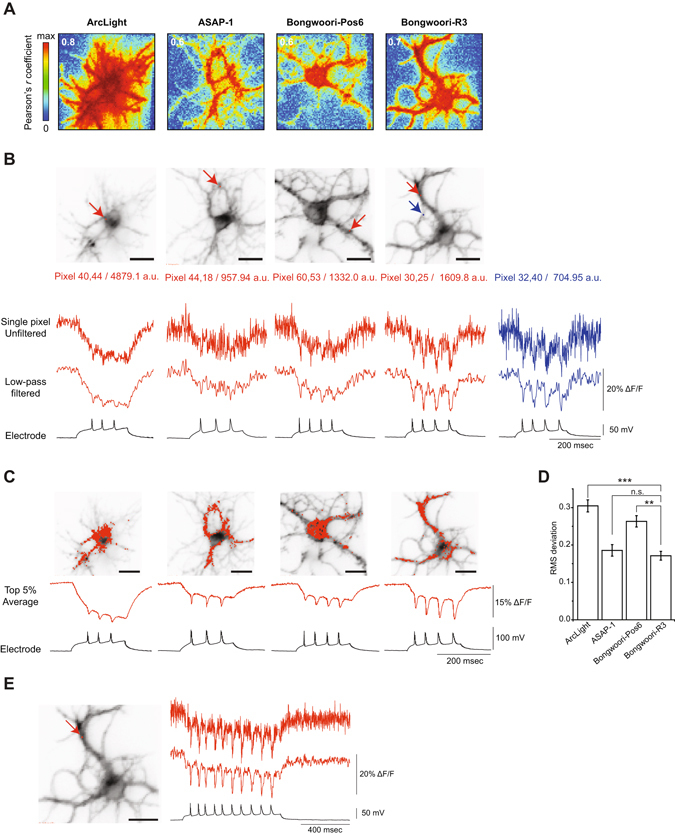
Single trial, single pixel traces exhibiting the highest correlation of fluorescence change to voltage. (**A**) Pearson’s correlation coefficient matrix of the fluorescence to the voltage. The highest coefficient value is indicated in white. (**B**) The red arrows indicate the position of the pixel with the highest correlation coefficient. The blue arrow indicates a pixel with lower fluorescence intensity. Numbers below each image refer to the fluorescence intensities (in arbitrary units, a.u.) of the selected pixels. Pixel signals are shown unfiltered (top) and low-pass filtered (middle). Electrical recording is shown for comparison (bottom). (**C**) Top 5% pixels with the highest correlation coefficient and corresponding average traces. (**D**) Root-mean square deviation of optical traces in (**C**) compared to the voltage signal. (**E**) Bongwoori-R3 signal from single pixel with the highest correlation coefficient. Low-pass Gaussian filter with cut-off frequency of 100 Hz was applied for filtering. Error bars are standard error of the mean. Asterisks in (**D**) indicate statistical significance (*p < 0.05, **p < 0.01, ***p < 0.001 and n.s.: not significant). Scale bars: 20 µm.

An emerging challenge for imaging voltage in slice or *in vivo* is the processing of the data collected. The ultimate goal is to no longer use electrodes and use only light for neuronal recordings. But how does one determine which pixels to choose for the regions of interest to be studied? To address this question, we first computed for each pixel the Pearson’s correlation coefficient of the fluorescence change to the voltage change from the best neurons expressing ArcLight, ASAP-1, Bongwoori-Pos6, or Bongwoori R3 (Fig. 7A). The pixel showing the highest coefficient was then extracted for each construct (Fig. 7B). Remarkably, an unfiltered trace from a single pixel for Bongwoori-R3 was still clearly able to resolve action potentials optically. Even a pixel from the Bongwoori-R3 expressing neuron with a lower light level, and therefore a lower SNR level, was able to resolve action potentials (Fig. 7B, blue trace and Table 4).

**Table 4:** Characteristics of GEVIs analyzed from highest correlated pixels.

Single pixels	ArcLight A242	ASAP1	Bongwoori-Pos6	Bongwoori-R3	Bongwoori-R3 (Two pixels with different brightness)	
					(30, 25)	(32, 40)
Brightness (a.u.)	2685 ± 390	1044 ± 31	1154 ± 46	1257 ± 130	1609.8	704.95
ΔF/F (%)	−19.7 ± 0.5	−10.8 ± 0.7	−10.7 ± 0.1	−15.4 ± 0.0	−17.1 ± 0.1	−15.0 ± 0.1
SNR	22.2 ± 1.6	7.3 ± 0.6	6.7 ± 1.1	9.6 ± 0.8	9.5 ± 0.6	8.3 ± 0.4

Two individual pixel data shown on the right are from Bongwoori-R3 traces in Fig. 7A (red and blue traces with different fluorescence intensities). All values were analyzed from low-pass filtered traces. Values were shown as mean ± SEM (standard error of the mean). The number of action potentials analyzed; ArcLight: 12, ASAP1: 12, Bongwoori: 11, Bongwoori-Pos6: 11, Bongwoori-R3: 12, all from three cells for each GEVI.

Expanding the region of interest to include the top 5% of pixels correlated to voltage improved the SNR (Fig. 7C). To quantify how these optical responses fit the electrical signals, we computed the root-mean-square deviation following amplitude normalizations (Fig. 7D). While ArcLight showed the largest signal size and SNR for evoked action potentials, ArcLight also exhibited the biggest deviation of the optical response to the voltage signal. Bongwoori-Pos6 showed a larger deviation of the optical signal to the voltage trace due to its left-shifted voltage sensitivity. The optical signals of the top 5% of correlated pixels from Bongwoori-R3 and ASAP-1 exhibited the best fits.

**Figure 8:**
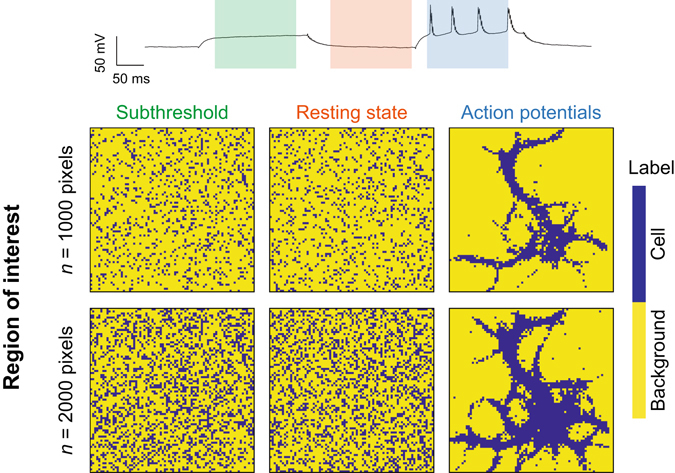
Defining regions of neuronal activity with an electrode-free method for Bongwoori-R3. Top, electrical recording of the neuron expressing Bongwoori-R3 from Fig. 7. Color-shaded boxes correspond to different time windows used for the correlation analyses. Bottom, regions of interest computed during the subthreshold, resting state, and action potentials for an arbitrary number *n* = 1000 pixels or *n* = 2000 pixels.

We next developed a method to define the region of interest that is independent of the electrical recording, taking advantage of the ability of single pixels (roughly one µm^2^ of the neuronal plasma membrane) to resolve action potentials. We propose a simple method based on pixel-to-pixel correlation of fluorescence traces to construct a mask of *n* arbitrarily chosen number of pixels and to define objectively the regions of interest (See methods). This correlation is similar to but distinct from that used for the GEVI, Arch^30^, which required a weighted matrix not needed for Bongwoori-R3. We first restrained this analysis for different time windows in the recording. Choosing the subthreshold and resting state yielded random ROIs, as expected by the correlation of noise traces, while the firing of action potentials accurately reconstructed the cell geometry (Fig. 8).

## Discussion

The GEVI, Bongwoori-R3, consists of three functionally distinct domains that participate in transforming changes in membrane potential into an optical signal. The VSD resides in the plasma membrane and responds to changes in voltage by altering its conformation. The linker domain couples the movement of the FP to the conformational change of the VSD. The FP domain provides the optical output. The FP domain has been shown to dimerize enabling the conformational change in the VSD to alter the environment of the chromophore thereby eliciting a fluorescent signal. Introduction of mutations that decrease the affinity of the FP to dimerize reduce the optical signal by over 70%^15^. Mutations to the VSD have improved the speed and the voltage range of the optical signal^4, 19^. In this report, we have shown that the charge composition of the linker can also affect the characteristics of the voltage-dependent optical signal offering GEVI developers multiple routes to improve the imaging of neuronal activity.

There are multiple explanations for how the linker domain affects the optical activity of the GEVIs reported here. One is that the linker can respond to voltage. Our attempt to test this hypothesis by limiting the movement of the VSD resulted in probes with a reduced optical signal when the linker was more negatively charged. When the linker was more positively charged, the voltage range of the probe was unaffected but the signal size increased (Fig. 1). These results suggested that the linker is important for orienting the FP and probably does not directly respond to changes in membrane potential. A previous report altering the amino acid composition at the FP fusion site as well as linker length also suggested the importance of orienting the FP^17^. In addition, the linker of the voltage-sensing phosphatase was shown to have a membrane binding site consisting of arginines that was important for coupling voltage to the enzymatic activity of the phosphatase^31^. The positive residue in the Bongwoori-R3 linker may play a similar role by associating with the phospholipid head groups of the lipid bilayer. The structure of the linker could also affect the motion of the FP. Since the negative charge on the outside of the β-barrel of the FP is responsible for the large optical signal of ArcLight^1^ and Pado^15^, the linker could also affect the path the negative charge travels during the movement of S4 which would also alter the fluorescent signal of the GEVI.

Bongwoori-R3 has more than doubled the size of the optical signal of the original Bongwoori giving signals as high as 20% ΔF/F per action potential in a single pixel. This improved signal is partially the result of a combination of the speed of the optical response and the voltage range of the probe. The voltage range of the probe determines the maximum fluorescence change the GEVI is capable of. The speed of the on tau of a GEVI predicts the percentage of that maximum fluorescence change the probe will exhibit. The speed of the off tau will provide a good estimate for the frequency of voltage change the GEVI can resolve. Therefore, slower probes are still capable of following action potentials albeit with a reduced signal strength from the potential maximum observed in HEK cells. ASAP-1 is faster than Bongwoori-R3, but Bongwoori-R3 gives a larger optical signal. The voltage sensitivity of the probe also plays a role dictating the ratio of the optical response of an action potential spike to that observed for subthreshold depolarizations (Figs 5, 6 and 7). It is the combination of these physical characteristics that determines a GEVI’s ability to optically resolve action potentials.

Bongwoori-R3 also exhibits a small change in the baseline fluorescence upon neuronal activation. This is primarily due to a change in the intracellular pH as indicated by the optical response of the farnesylated FP (SE227D) in Fig. 6. Interestingly, the baseline change was more pronounced in the processes than in the soma presumably due to the soma’s larger volume and therefore higher buffering capacity. The change in intracellular pH does not seem to affect Bongwoori-R3′s ability to optically resolve action potentials. Indeed, this also enables the detection of acidification during single cell imaging. A similar baseline effect was seen for ArcLight (Fig. 6).

There are many GEVIs available to the scientific community (recently reviewed in refs 2, 3, 32, 33). All have different strengths and weaknesses. Here, we describe a new version of Bongwoori, Bongwoori-R3, that gives a large change in fluorescence at speeds capable of resolving action potentials at 65 Hz (Fig. 5D). Bongwoori-R3 also gives a robust optical signal during 2-photon imaging (Fig. 4E). The improved optical signal of Bongwoori-R3 enables the reconstruction of the neuron using only fluorescence changes. Plotting pixels with correlated fluorescence changes resulted in the reconstruction of the neuron based on neuronal activity. By choosing appropriate frames for correlation, the pixels exhibiting neuronal activity can be identified. For single cell imaging this does not provide new information since one could just choose the pixels representing the cell based on the resting light fluorescence. However, for studying populations of cells, this ability to determine correlation based only on fluorescence change will help in the analysis of neuronal circuits. Up until now, most regions of interest for the analysis of optical signals have been determined arbitrarily. Having probes capable of larger fluorescence changes, which equates to neuronal activity, allows pixel correlation analyses to remove the experimenter’s bias in determining where the location of the neuronal activity is.

## Electronic supplementary material

Supplementary Information
