# Supplementary material for: Improving a genetically encoded voltage indicator by modifying the cytoplasmic charge composition
Source: Sci Rep. 2017 Aug 15;7:8286. doi: 10.1038/s41598-017-08731-2 (PMC5557843; doi:10.1038/s41598-017-08731-2)
Supplement: Supplementary file 1 — Supplementary Information [file 41598_2017_8731_MOESM1_ESM.pdf]

Improving a genetically-encoded voltage indicator by modifying the cytoplasmic charge composition

- Sungmoo Lee[1,2], Tristan Geiller[1,3], Arong Jung[1,4], Ryuichi Nakajima[1], Yoon-Kyu Song[2,5] and Bradley J. Baker[1,6]

1. The Center for Functional Connectomics, Korea Institute of Science and Technology, Seoul, South Korea
2. Program in Nanoscience and Technology, Graduate School of Convergence Science and Technology, Seoul National University, Suwon, South Korea
3. Department of Psychology, Korea University, Seoul, South Korea
4. College of Life Sciences and Biotechnology, Korea University, Seoul, South Korea
5. Advanced Institutes of Convergence Technology, Suwon, South of Korea
6. Division of Bio-Medical Science and Technology, KIST School, Korea University of Science and Technology (UST), Seoul 02792, Republic of Korea

Correspondence should be addressed to Dr. Bradley Baker; bradley.baker19@gmail.com

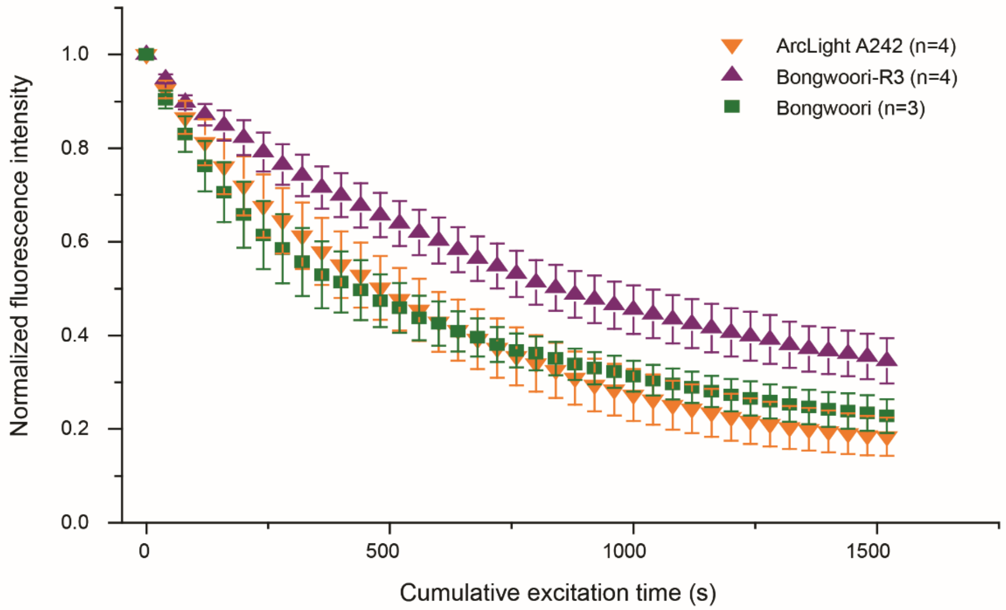

Supplementary figure 1. Normalized fluorescence intensities of three GEVIs showing their photobleaching rates in HEK 293 cells. ArcLight A242, Bongwoori or Bongwoori-R3 expressing cells were excited with a Xenon arc lamp by repeating a 40s light-on / 20s light-off trial. Fluorescence intensities were measured every 40s and fitted to an exponential decay function. Time constants for all three GEVIs were longer than 450s. Excitation light intensity was 1 mW/mm^2.

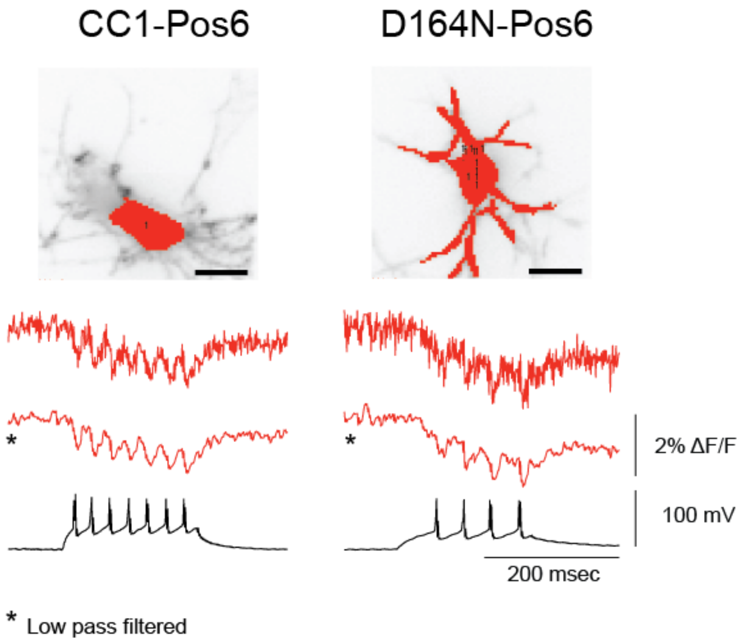

Supplementary figure 2. Optical imaging of evoked action potentials from cultured mouse hippocampal neurons with two untuned GEVIs, CC1-Pos6 and D164N-Pos6. For low-pass filtration, Gaussian filter was used. Upper trace is unfiltered and lower trace is low-pass filtered. Scale bar = 20μm.

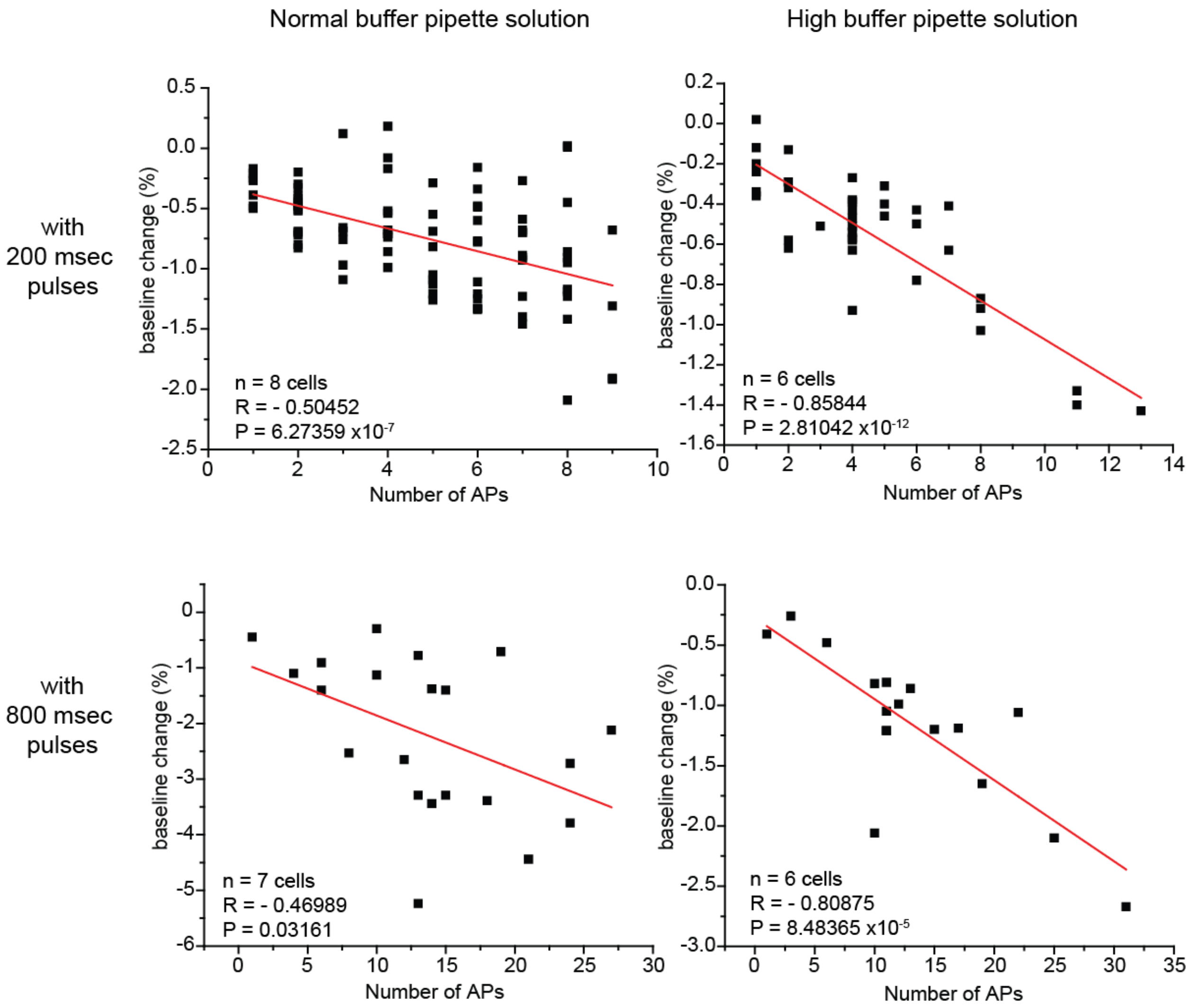

Supplementary figure 3. Baseline change in ΔF/F measured with Bongwoori-R3 correlates with the number of evoked action potentials. The amplitude of baseline change of soma was plotted against the number of evoked action potentials. Pipette solution with normal buffering capacity (5 mM HEPES) or with high buffering capacity (100 mM HEPES) were used and plotted separately.

Primer	Sequence	Construct
NEG1-A	GAATATTTGACTCCCACCAAGAGATGGGGGATCCCATGAG	CC1-Neg1
NEG1-B	GGGATCCCCCATCTCTTGGTGGGAGTCAAATATTCTTGC	
NEG2-A	GAATATTTGATTCCCACGAGGAAATGGGGGATCCCATGAG	CC1-Neg2
NEG2-B	GGGATCCCCCATTTCCTCGTGGGAATCAAATATTCTTGC	
NEG3-A	GAATATTTTATGACCACCAAGAGATGGGGGATCCCATGAG	CC1-Neg3
NEG3-B	GGGATCCCCCATCTCTTGGTGGTCATAAAATATTCTTGC	
NEG-4AA	CAAGAATATTTTATGATCACGAACACGAGGGGGATCCCATGAGTAAAGG	CC1-Neg4
NEG4-BB	CCTTTACTCATGGGATCCCCCTCGTGTTCGTGATCATAAAATATTCTTG	
POS5-A	GAATATTTAGGTCCCACCAAAGAATGGGGAGGCCTATGAG	CC1-Pos5
POS5-B	AGGCCTCCCCATTCTTTGGTGGGACCTAAATATTCTTGC	
POS6-A	GAATATTTAGATCCCACAGAAGGATGGGGAGGCCTATGAG	CC1-Pos6
POS6-B	AGGCCTCCCCATCCTTCTGTGGGATCTAAATATTCTTGC	
POS7-A	GAATATTTTATAGACACCAACGGATGGGGAGGCCTATGAG	CC1-Pos7
POS7-B	AGGCCTCCCCATCCGTTGGTGTCTATAAAATATTCTTGC	
POS8-AA	GCAAGAATATTTTATTCCAGGCAACAAAGGGGCAGGCCTATGAGTAAAGGAGAAG	CC1-Pos8
POS8-BB	CTTCTCCTTTACTCATAGGCCTGCCCCTTTGTTGCCTGGAATAAAATATTCTTGC	
SM014A	GGTTAGCAAGAATATTTTATTCCCACCAACAAATGGGGGATCCCATGAG	CC1-M240
SM014B	CTCATGGGATCCCCCATTTGTTGGTGGGAATAAAATATTCTTGCTAACC	
SM020A	GCTGCGTGTGGTTATCTTAGCAAGAATATTTAGATCCCACAGAAGG	Bongwoori-Pos6
SM020B	AAATATTCTTGCTAAGATAACCACACGCAGCAATCTGGCCAACAC	
SM029A	CTTGTTATTTCATGCTGAATTTAGGATTAAGGATATTTGCC	D164N-Pos6
SM029B	GGCAAATATCCTTAATCCTAAATTCAGCATGAAATAACAAG	
SM042A	TTTAAATCCCACAAAAAGATGGGGAAGCCTATGAGTAAAGGAG	CC1-Pos6-Ks
SM042B	GGCTTCCCCATCTTTTTGTGGGATTTAAATATTCTTGCTAACCG	
SM030A	CGCCGGAGAAGGCGGAGAAGGCGCATGAGTAAAGGAGAAGAACTTTTC	CC1-9Rs
SM030B	GCGCCTTCTCCGCCTTCTCCGGCGTCTAAATATTCTTGCTAACCGAACC	
SM048A	GAAAAAGAAGAAAAAGAAAAAGAAGATGAGTAAAGGAGAAGAACTTTTC	CC1-9Ks
SM048B	CTTCTTTTTCTTTTTCTTCTTTTTCTTAAATATTCTTGCTAACCGAACC	
SM049A	GGAAGAAGAGGAAGAGGAGGAAGAAATGAGTAAAGGAGAAGAACTTTTC	CC1-9Es
SM049B	TTCTTCCTCCTCTTCCTCTTCTTCCTCAAATATTCTTGCTAACCGAACC	
SM050A	CTCATCTTCCTCATCCTCTTCCTCAATGAGTAAAGGAGAAGAACTTTTC	CC1-9Ss
SM050B	TGAGGAAGAGGATGAGGAAGATGAGGAAAATATTCTTGCTAACCGAACC	
SM051A	GCAGCAACAGCAGCAACAGCAGCAAATGAGTAAAGGAGAAGAACTTTTC	CC1-9Qs
SM051B	TTGCTGCTGTTGCTGCTGTTGCTGCTGAAATATTCTTGCTAACCGAACC	
SM055A	GATGACGATGATGACGATGATGACATGAGTAAAGGAGAAGAACTTTTC	CC1-9Ds
SM055B	ATCATCGTCATCATCGTCATCATCAAATATTCTTGCTAACCGAACCAC	
SM056A	GCTGCCGCAGCTGCCGCAGCTGCCATGAGTAAAGGAGAAGAACTTTTC	CC1-9As
SM056B	AGCTGCGGCAGCTGCGGCAGCGGCAAATATTCTTGCTAACCGAACCAC	
BR1A	GGTTATCTTAGCAAGAATATTTAGGTCCCACCAACAAGGGG	Bongwoori-R1
BR1B	CCCCTTGTTGGTGGGACCTAAATATTCTTGCTAAGATAACC	
BR2A	CTTAGCAAGAATATTTTATAGGCACCAACAAGGGGATCCC	Bongwoori-R2
BR2B	GGGATCCCCTTGTTGGTGCCTATAAAATATTCTTGCTAAG	
BR3A	GCAAGAATATTTTATTCCAGGCAACAAGGGGATCCCATGAG	Bongwoori-R3
BR3B	CTCATGGGATCCCCTTGTTGCCTGGAATAAAATATTCTTGC	
BR4A	GAATATTTTATTCCCACAGGCAAGGGGATCCCATGAGTAAAGG	Bongwoori-R4
BR4B	CCTTTACTCATGGGATCCCCTTGCCTGTGGGAATAAAATATTC	
BR5A	GAATATTTTATTCCCACCAAAGGGGGGATCCCATGAGTAAAGG	Bongwoori-R5
BR5B	CCTTTACTCATGGGATCCCCCCTTTGGTGGGAATAAAATATTC	
BR6A	GAATATTTTATTCCCACCAACAAAGGGATCCCATGAGTAAAGG	Bongwoori-R6
BR6B	CCTTTACTCATGGGATCCCTTTGTTGGTGGGAATAAAATATTC	
BR7A	GAATATTTTATTCCCACCAACAAGGGAGGCCCATGAGTAAAGGAG	Bongwoori-R7
BR7B	CTCCTTTACTCATGGGCCTCCCTTGTTGGTGGGAATAAAATATTC	
BR8A	CAACAAGGGGATAGGATGAGTAAAGGAGAAGAACTTTTCACTGG	Bongwoori-R8
BR8B	CCTTTACTCATCCTATCCCCTTGTTGGTGGGAATAAAATATTC	

Supplementary table 1. Primer sequences used for PCR reactions to generate DNA gene constructs in this work.
